# Coexistence of papilledema and pseudopapilledema after remission of idiopathic intracranial hypertension by bariatric surgery

**DOI:** 10.5935/0004-2749.20200030

**Published:** 2020

**Authors:** Luciano Mesquita Simão, Thaís Nunes Andrade, Ana Flávia Alves de Oliveira e Oliveira, Andressa Guimarães Aguilar Garcez, Raíza Jacometti

**Affiliations:** 1 Serviço de Neuro-Oftalmologia, Instituto de Olhos, Faculdade de Ciências Médicas de Minas Gerais, Belo Horizonte, MG, Brazil; 2 Instituto de Olhos, Faculdade de Ciências Médicas de Minas Gerais, Belo Horizonte, MG, Brazil

**Keywords:** Papilledema, Optic disk drusen, Intracranial hypertension, Magnetic resonance imaging, Bariatric surgery, Humans, Case reports, Papiledema, Drusas do disco óptico, Hipertensão intracraniana, Imagem por ressonância magnética, Cirurgia bariátrica, Humanos, Relatos de casos

## Abstract

A 37-year-old woman complained of headaches following bilateral visual loss in the past two years. She was obese and had undergone bariatric surgery three months earlier, followed by a considerable weight loss. Neuro-ophthalmic exa mination revealed a bilateral swollen optic disk. After a computerized analysis of the visual fields and magnetic resonance imaging of the brain and orbits, a diagnosis of idiopathic intracranial hypertension was made. At six months after the bariatric surgery, the patient reported no further headaches and exhibited better findings on computerized analysis of visual fields. However, fundus examination revealed persistent mild papilledema in both eyes. Ocular B-scan ultrasonography showed bilateral optic disk drusen. This report highlights the coexistence of true papilledema and pseudopapilledema due to optic disk drusen, following remission of idiopathic intracranial hypertension after a bariatric surgery.

## Abstract (pt)

Uma mulher de 37 anos queixou-se de cefaleia após perda visual bilateral nos últimos dois anos. Apresentava história de obesidade e havia sido submetida à cirurgia bariátrica três meses antes, seguida de considerável perda de peso. O exame neuro-oftálmico revelou um disco óptico inchado bilateral. Após uma análise computadorizada dos campos visuais e ressonância magnética do crânio e órbitas, foi feito um diagnóstico de hiper tensão intracraniana idiopática. Após seis meses da cirurgia bariátrica, a paciente não relatou mais cefaleia e foram descober tas melhoras na análise computadorizada dos campos visuais. No entanto, o exame de fundo de olho revelou papiledema leve persistente em ambos os olhos. A ultrassonografia ocular B-scan mostrou drusas do disco óptico bilateralmente. Este relato desta ca a coexistência de papiledema verdadeiro e pseudopapiledema devido à drusa de disco óptico após remissão da hipertensão intracraniana idiopática após uma cirurgia bariátrica.

## INTRODUCTION

Idiopathic intracranial hypertension (IIH) is a headache syndrome characterized by increased cerebrospinal fluid (CSF) pressure in the absence of an intracranial mass lesion, with normal CSF composition. In this condition, neurologic examinations generally show normal results, except for papilledema and occasional cranial nerve VI palsy^(1)^.

In adults, the incidence of cerebral pseudotumor syndrome is estimated at 1-2 out of 100,000 people. Women, particularly those who are obese and of childbearing age, are more susceptible to the development of cerebral pseudotumor syndrome^(2)^. The most common symptom of cerebral pseudotumor syndrome is headache, which is reported by most patients as the primary symptom. This syndrome is marked by visual changes, which constitute a presenting symptom for many patients. Transient visual obscurations are likely in most patients (68%) and may occur both unilaterally and/or bilaterally. Diplopia is also reported, but more rarely^(3)^.

Optic disk drusen are important in the differential diagnosis of cerebral pseudotumor syndrome, as they represent the main cause of pseudopapilledema. Optic disk drusen are abnormal accumulations of calcified mitochondrial deposits in the optic nerve head^(4)^. This phenomenon occurs in 0.3%-2.0% of the population and is bilateral in 75% of the cases. Patients are generally asymptomatic; however, changes in the visual acuity and visual fields may occur in cases of nonarteritic anterior ischemic optic neuropathy associated with optic disk drusen, especially in young patients^(5)^.

Drusen are often considered to be congenital, and var ious mechanisms have been proposed to explain their formation. A current theory is that a small scleral canal can cause axonal stress because of the physical limitations of the large number of axons of the optic nerve in a small space. A second theory is that drusen formation could occur as a result of abnormal vascularization of the optic disk, with consequent ischemia, stress, and weakened axonal metabolism^(6)^.

In this report, we describe the case of a 37-year-old woman with a clinical history and examination suggestive of IIH. However, the persistent fundus appearance due to papilledema confirmed the coexistence of optic disk drusen.

## CASE REPORT

**Figure 1:**
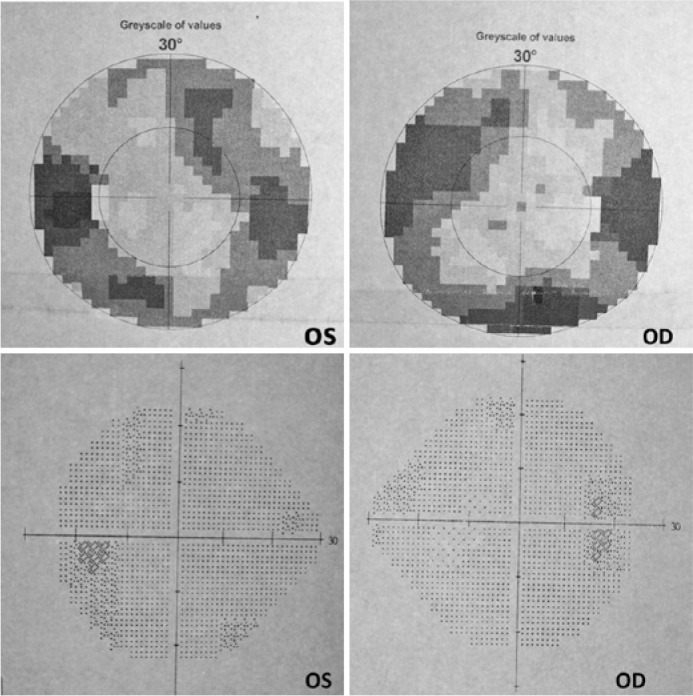
Computerized visual field analysis showing the loss of peripheral visual fields in both eyes (above) and normal findings (below).

**Figure 2:**
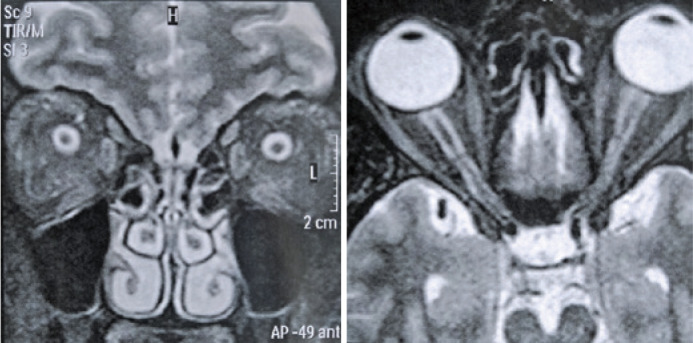
MRI of the brain and the orbits showing flattening of the globe, an enlarged optic nerve sheath, protrusion and enhancement of the optic nerve head, and vertical buckling of the optic nerve.

**Figure 3:**
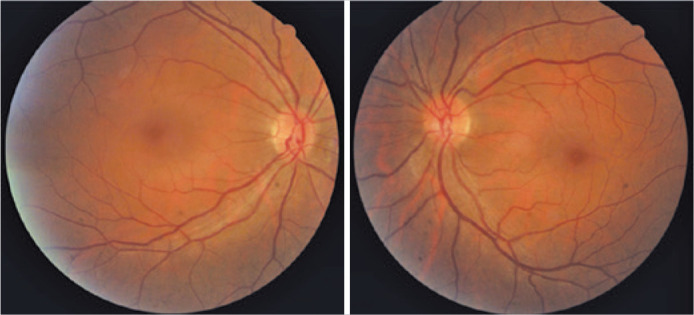
Fundoscopy revealing persistent mild papilledema in both eyes.

**Figure 4:**
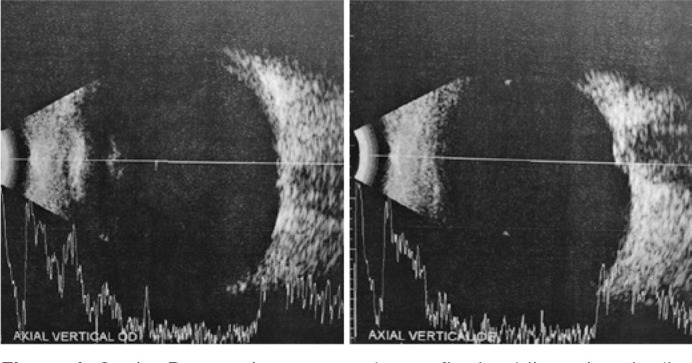
Ocular B-scan ultrasonography confirming bilateral optic disc drusen.

A 37-year-old woman complained of headaches following bilateral visual loss in the past two years, which had become more frequent in the past six months. She was obese and had undergone bariatric surgery three months earlier, followed by a considerable weight loss. In her first neuro-ophthalmic examination, she had visual acuity of 20/20 in both eyes; her pupils were equal in size and no afferent pupillary defects were present. Version was full and no other abnormalities were found on examination except the fundus finding of a bilateral swollen optic disk. Computerized analysis of the visual fields showed loss of the peripheral visual field in both eyes (Figure 1). Magnetic resonance imaging (MRI) of the brain and the orbits revealed flattening of the globe, an enlarged optic nerve sheath, protrusion and enhancement of the optic nerve head, and vertical buckling of the optic nerve (Figure 2). Lumbar puncture, including raquimanometry, was not available; however, our patient had objective signs of IIH, such as holocranial headache, obesity, tinnitus, and papilledema. The diagnosis of IIH was made with the presumption of good prognosis of visual function after the bariatric surgery. At six months after the bariatric surgery, the patient had no further headaches, 20/20 vision bilaterally, and better visual field findings on computerized analysis (Figure 1). However, the fundus examination revealed persistent mild papilledema in both eyes (Figure 3). Ocular B-scan ultrasonography showed optic disk drusen bilaterally (Figure 4).

## DISCUSSION

Historically, there has been a known association between cerebral pseudotumor syndrome and obesity; however, the specific underlying physiopathology remains unknown. A proposed mechanism is that obesity elevates the intra-abdominal pressure, thereby increasing the pleural and cardiac pressure; this could lead to a reduction of venous return, causing reduced reabsorption of fluids. The relationships between obesity and high cytokine, interleukin, and leptin levels also suggest an inflammatory component^(2)^.

Bariatric surgery seems to reduce the adverse effects of cerebral pseudotumor syndrome through substantial weight loss^(7)^. In recent years, this option has gained momentum because of its multiple benefits for the health of morbidly obese patients. However, the current literature consists solely of case reports or small retrospective studies suggesting that bariatric surgery is an effective treatment for cerebral pseudotumor syndrome^(8)^.

Birnbaum et al. reported that the prevalence of optic disk drusen was 19% in patients with resolution of papilledema due to intracranial hypertension, which was significantly higher than the expected rate in the general population^(6)^. No causal connection could be de termined from the results of that study, as the effect of time was not established; notably, no patients with disk drusen were evaluated prior to the development of intracranial hypertension and papilledema.

Papilledema, specifically the optic disk edema due to increased intracranial pressure, is known to produce axoplasmic stasis because of the mechanical obstruction of axons, ischemia, or both; thus, papilledema could eventually promote the formation of optic disk drusen. In contrast, a plausible biological mechanism for an increased risk of papilledema in eyes with disk drusen can be justified by the reduction of the space available for axonal edema of the nerve, due to the presence of optic disk drusen^(6)^.

Our patient presented objective signs of IIH, such as headache, obesity, tinnitus, papilledema, and presumed coexistence of pseudopapilledema due to bilateral optic disk drusen. This striking case highlights the coexis tence of true papilledema and pseudopapilledema due to optic disk drusen in a patient who experienced a considerable weight loss after bariatric surgery and exhibited a persistent swollen optic disk after recovery of visual function. It is merely speculative whether the optic disk drusen were present before the papilledema in our patient.
